# Parliament and the Profession

**Published:** 1918-10-26

**Authors:** 


### Parliament and the Profession.

The House of Commons resumed after the adjournment on October 15.

### Midwives Bill.

The Midwives Bill was read a second time on October 15.

### Venereal Diseases.

The Home Secretary dealt with several questions in reference to Defence of the Realm Regulation 40 D (which provides for the prosecution of women knowingly running the risk of communicating venereal disease to soldiers). Sir George Cave said that there had been 201 prosecutions under the Regulation. Convictions had been obtained in 102 cases, in fifty-one of which the defendants pleaded guilty; he said that the return indicated that the magistrates are very careful, and do not convict except in very clear cases. The Regulation is being considered by a committee, who have been asked to report as early as possible. This committee will consider the Regulation, and what modifications (if any) should be made in its terms or in the procedure for enforcing it, and would not be precluded from considering the question of its withdrawal.

### Treatment of Discharged Soldiers.

Mr. Bonar Law dealt with several questions addressed to the Prime Minister raising a doubt as to the adequacy of the arrangements for the treatment and training for discharged soldiers and suggesting the appointment of a committee to inquire into the reason of the alleged failure. Mr. Bonar Law was satisfied that all that is possible is being done by the Pensions Ministry, and he did not agree with the necessity of appointing a committee.

